# Extracellular vesicles for acute kidney injury in preclinical rodent models: a meta-analysis

**DOI:** 10.1186/s13287-019-1530-4

**Published:** 2020-01-03

**Authors:** Chao Liu, Jin Wang, Jie Hu, Bo Fu, Zhi Mao, Hengda Zhang, Guangyan Cai, Xiangmei Chen, Xuefeng Sun

**Affiliations:** 1 Department of Nephrology, Chinese PLA General Hospital, Chinese PLA Institute of Nephrology, State Key Laboratory of Kidney Diseases, National Clinical Research Center for Kidney Diseases, 28 Fuxing Road, Beijing, China; 2 Department of Critical Care Medicine, Chinese PLA General Hospital, 28 Fuxing Road, Beijing, China; 3 Master Program of Medical Science in Clinical Investigation, Harvard Medical School, 25 Shattuck Street, Boston, MA USA

**Keywords:** Extracellular vesicles, Exosomes, Mesenchymal stromal cells, Acute kidney injury, Meta-analysis

## Abstract

**Introduction:**

Extracellular vesicles (EVs), especially stem cell-derived EVs, have emerged as a potential novel therapy for acute kidney injury (AKI). However, their effects remain incompletely understood. Therefore, we performed this meta-analysis to systematically review the efficacy of EVs on AKI in preclinical rodent models.

**Methods:**

We searched PubMed, EMBASE, and the Web of Science up to March 2019 to identify studies that reported the treatment effects of EVs in a rodent AKI model. The primary outcome was serum creatinine (Scr) levels. The secondary outcomes were the blood urea nitrogen (BUN) levels, renal injury score, percentage of apoptotic cells, and interleukin (IL)-10 and tumour necrosis factor (TNF)-α levels. Two authors independently screened articles based on the inclusion and exclusion criteria. The meta-analysis was conducted using RevMan 5.3 and R software.

**Results:**

Thirty-one studies (*n* = 552) satisfied the inclusion criteria. Pooled analyses demonstrated that the levels of Scr (SMD = − 3.71; 95% CI = − 4.32, − 3.10; *P* < 0.01), BUN (SMD = − 3.68; 95% CI = − 4.42, − 2.94; *P* < 0.01), and TNF-α (SMD = − 2.65; 95% CI = − 4.98, − 0.32; *P* < 0.01); the percentage of apoptotic cells (SMD = − 6.25; 95% CI = − 8.10, − 4.39; *P* < 0.01); and the injury score (SMD = − 3.90; 95% CI = − 5.26, − 2.53; *P* < 0.01) were significantly decreased in the EV group, and the level of IL-10 (SMD = 2.10; 95% CI = 1.18, 3.02; *P* < 0.01) was significantly increased. Meanwhile, no significant difference was found between stem cell-derived EVs and stem cells.

**Conclusion:**

The present meta-analysis confirmed that EV therapy could improve renal function and the inflammatory response status and reduce cell apoptosis in a preclinical rodent AKI model. This provides important clues for human clinical trials on EVs.

## Background

Acute kidney injury (AKI) is a major kidney disease characterised by a rapid decline in renal function and is associated with an increase in mortality and hospitalisation [1]. However, the prognosis of this disease, which may occur under various circumstances, has not been significantly improved since the mid-1990s [2]. Due to the lack of efficient therapeutic methods, patients with renal ischaemia reperfusion injury (IRI) are mostly treated by supportive manoeuvres, such as renal replacement therapy [3].

Many studies have confirmed that mesenchymal stem cell (MSC) therapy can effectively improve AKI [4, 5], but most of these studies have not found that MSCs colonise in the kidneys to play a direct role [4, 6]. Moreover, MSC therapy may have certain risks, such as inducing tumours, and its safety remains questionable [7].

Recently, data in the literature have highlighted that the delivery of MSC-derived EVs can ameliorate AKI in preclinical models [3, 6, 8]. EVs are secreted by almost all types of cells and can be subdivided into exosomes, microvesicles, and apoptotic bodies [9]. Exosomes are the smallest vesicles (30–100 nm) released by the fusion of multivesicular bodies containing intraluminal vesicles with the plasma membrane. Microvesicles are vesicular structures (0.1–1.0 μm) shed by outward blebbing of the plasma membrane. The largest EVs (1–5 μm) are apoptotic bodies that are formed during the late stages of apoptosis [10]. EVs contain proteins, lipids, carbohydrates, mRNAs, and miRNAs and may influence different cell types acting on physiological processes such as proliferation and immune escape [11]. Compared with MSCs, the small size of MSC-derived EVs allows them to avoid the pulmonary first-pass effect and to penetrate deep inside most body barriers [3]. Therefore, MSC-derived EVs are expected to be an effective treatment for AKI.

Many animal studies have been performed to investigate the efficacy of EVs on an AKI model with various cell origins and different injection doses, delivery routes, and therapy times [3, 12]. To provide the most recent available evidence for clinical studies, we performed this meta-analysis to investigate the efficacy of EVs on preclinical rodent models.

## Materials and methods

Preferred Reporting Items for Systematic Reviews and Meta-Analyses (PRISMA) was used to perform this meta-analysis [13].

### Search strategy

We searched PubMed, EMBASE, and the Web of Science from database inception to March 2019. The search terms were as follows: (“extracellular vesicles” or “EVs” or “micro vesicles” or “micro-vesicles” or “microvesicles” or “microparticle” or “exosome” or “MVs” or “shedding vesicles”) and (“AKI” or “acute kidney injury” or “renal ischaemia-reperfusion” or “acute renal failure”). The search was limited to rodent models with no language restrictions. The reference lists of selected studies were searched by hand to identify potentially relevant citations. Ethical approval was not required because the meta-analysis was based on published articles.

### Study selection

Two independent investigators (CL and JW) conducted the study selection. Disagreements between the investigators were resolved in meetings or adjudicated by a third reviewer (XS).

### Eligibility criteria

The inclusion criteria were as follows: (1) population—rodent models with AKI; (2) intervention—various cell-derived EVs; (3) comparison—placebo; and (4) outcome measure—the primary outcome was the level of serum creatinine (Scr). The secondary outcomes were the renal injury score, percentage of apoptotic cells, and levels of blood urea nitrogen (BUN), interleukin (IL)-10, and tumour necrosis factor (TNF)-α.

The exclusion criteria were as follows: (1) AKI was not performed on rodent models, (2) repeated data, (3) insufficient information, and (4) review, letter, commentary, correspondence, case report, conference abstract, expert opinion, or editorial.

### Data extraction

Data extraction was performed by two independent reviewers (CL and JH) using a standardised form. The following data were collected: first author, country or region, publication year, number of animals, type of AKI model, species, treatment time, measurement time and EV cell origins, diameter, and dose. For studies that had not shown the corresponding results, Engauge Digitizer version 4.1 software was used to extract data from the graphics [14, 15].

### Quality assessment

The methodological quality of each included study was evaluated by two independent authors (JW and ZM) with a Collaborative Approach to Meta-Analysis and Review of Animal Data from Experimental Studies (CAMARADES) 10-item checklist [16]: A, peer-reviewed journal; B, temperature control; C, animals were randomly allocated; D, blind established model; E, blinded outcome assessment; F, use of anaesthetic without significant intrinsic vascular protection activity; G, appropriate animal model (diabetic, advanced age, or hypertensive); H, calculation of the sample size; I, statement of compliance with animal welfare regulations; and J, statement of potential conflicts of interest.

### Statistical analysis

All statistical analyses were conducted using RevMan version 5.3 and R statistical software version 3.4.1. Statistical significance was set at *P* < 0.05 (two-tailed). Continuous outcomes are expressed as the standardised mean difference (SMD) with the 95% CI. Heterogeneity was analysed among studies using the *I*^2^ statistic. *I*^2^ > 50% indicated significant heterogeneity [17]. Subgroup, sensitivity, and meta-regression analyses were performed to investigate potential between-study heterogeneity and to explore other potentially confounding factors. A cumulative meta-analysis was performed to explore changes in the results over time. Funnel plots and Egger’s test were conducted to detect publication bias. If publication bias was indicated, we further evaluated the number of missing studies by the Trimfill method and recalculated the pooled risk estimation with the addition of those missing studies.

## Results

### Search results and study characteristics

**Fig. 1:**
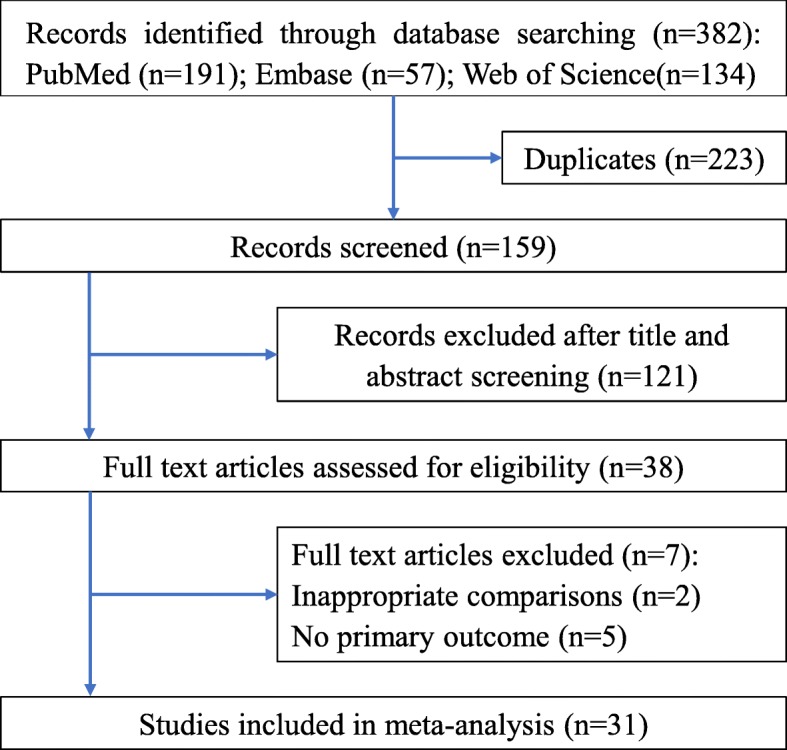
Flow chart of the study selection

The process of study selection is outlined in Fig. 1. In total, 31 studies satisfied the inclusion criteria [18–48]. The main characteristics of the included studies are presented in Table 1. All these studies were published between 2009 and 2019, and a total of 552 rodent animals were included in this meta-analysis. Among the included studies, 8 used bone marrow mesenchymal stromal cell (BMSC)-EVs [18, 27, 32, 40, 46–48], 6 used human umbilical cord mesenchymal stromal cell (UCMSC)-EVs [24, 28, 29, 36, 43, 44], 4 used human umbilical Wharton’s jelly mesenchymal stromal cell (WJMSC)-EVs [20, 30, 34, 38, 39], 3 used human umbilical vein endothelial cell (UVFC)-EVs [21, 31, 37], 2 used kidney-derived mesenchymal stromal cell (KMSC)-EVs [25, 42], 2 used adipose-derived mesenchymal stromal cell (ADMSC)-EVs [33, 35], 1 used human liver stem cell (HLSC)-EVs [41], and the 5 remaining used another origin of EVs [19, 22, 23, 26, 45]. The AKI model was established with IRI [18, 20–23, 25, 26, 28–34, 36–40, 42, 44, 45, 47], cisplatin [24, 35, 43, 46], glycerol [27, 41, 48], or the caecal ligation and puncture (CLP) method [19]. The diameter of the isolated EVs ranged from 15 to 1000 nm (mostly 50–200 nm). Surface markers, including CD63, CD9, CD81, and tumour susceptibility gene (TSG) 101, were used to identify and sort EVs from other components. A variety of microRNAs have been reported in EVs, such as miR-21 [19], miR-451 [27], miR-486-5p [31], miR-30 [34], and miR-199a-5p [18]. Most studies injected 100 μg EVs intravenously after the injury model was established [20, 22, 23, 26, 28–30, 33–35, 38–40, 46] (Table 1).

**Table 1:** Characteristics of included studies

Study	Country or region	Injury type	Species	Sex	Number	Cell source of EVs	Diameter (nm)	Administration methods	Therapy time	Measurement time	Dose	Main finding
Wang et al. [18]	China	IRI (45 min, bilateral)	BALB/c mice	Male	C: 5 T: 5	BMSC	120.6 (40–150)^b^	Tail vein	1 h prior to IRI	8, 16, 24, and 48 h after reperfusion	5 × 10^10^ particles in 100 μL	The administration of BMSC exosomes at the very early reperfusion stages significantly protected against renal I/R injury, and ER stress was closely linked to this protection.
Pan et al. [19]	China	CLP	C57BL/6 mice	Male	C: 4 T: 4	Derived from rIPC mice	15–150	Tail vein	After CLP	24 h	30 μg	Demonstrated a critical role for exosomal mir-21 in renoprotection conferred by limb rIPC against sepsis and suggested that rIPC and exosomes might serve as possible therapeutic strategies for sepsis-induced kidney injury.
Wu et al. [20]	China	IRI (cardiac arrest induced then transplant)	SD rats	Male	C: 40 T: 40	WJMSC	NR	Tail vein	After renal transplantation	24 h, 48 h, 1 and 2 weeks	100 μg in 1 mL	The administration of MVs immediately after renal transplantation ameliorated IRI in both the acute and chronic stages.
Vinas et al. [21]	Canada	IRI (30 min, bilateral)	FVB mice	Male	C: 6 T: 4	UVEC	88	Tail vein	After reperfusion	24 h	20 μg in 100 μL	UVEC exosomes selectively targeted the kidneys after ischaemic injury, with rapid cellular transfer of mir-486-5p. Targeting exosomes may involve the interaction between CXCR4 and endothelial cell SDF-1α.
Dominguez et al. [22]	USA	IRI (50 min, bilateral)	Nude rats	NR	C: 4 T: 5	Human renal tubular cells	115 ± 0.9^a^	Tail vein	2 days after reperfusion	1–6 days	100 μg	Renal damage from severe ischaemia was broad, and human renal exosomes prevented most protein alterations. Exosomes seem to acutely correct a critical and consequential abnormality during reperfusion.
Zhang et al. [23]	China	IRI (45 min on left kidney, remove right kidney)	SD rats	Male	C: 6 T: 6	Ischaemic preconditioned kidney serum	225 ± 83.2^a^ (150–350)	Intravenous	After reperfusion	24 h	100 μg	Remote ischaemic preconditioning played a therapeutic role in renal IRI through EVs induced by hypoxia.
Wang et al. [24]	China	Cisplatin (5 mg/kg, 3 days)	SD rats	NR	C: 6 T: 6	UCMSC	Peaking at 102	Renal capsule	0.5 h before cisplatin administration	24 h, 48 h, 72 h	200 μg	UCMSC-derived exosomes prevented against cisplatin-induced AKI through an autophagy-related mechanism. Therefore, pretreatment with UCMSC-Ex may be a new method to improve the therapeutic effect of cisplatin.
Ranghino et al. [25]	Italy	IRI (35 min on left kidney, remove right kidney)	SCID mice	Male	C: 6 T: 6	Gl-MSC-EVs	170 ± 62^a^	Tail vein	After reperfusion	48 h	4 × 10^8^ particles	Gl-MSCs might contribute to kidney repair after ischaemic AKI. The mechanism can, at least in part, be ascribed to the release of EVs that are able to mimic the effect of Gl-MSCs.
Dominguez et al. [26]	USA	IRI (50 min, bilateral)	SD rats	Female	C: 5 T: 5	Renal tubular cells	100 ± 3.94^a^	Tail vein	24 h and 48 h	1–6 days	100 μg in 0.5 mL	Treatment with EVs from adult renal cells applied well after IRI improved multiple structure and function parameters and transcriptome profiles.
Bruno et al. [27]	Italy	Glycerol (8 mL/kg, 3 days)	SCID mice	NR	C: 10 T: 10	BMSC	160 ± 72^a^	Tail vein	3 days after glycerol injection	48 h	165 × 10^6^ particles	The different molecular compositions of exosome- and microvesicle-enriched populations may explain the regenerative effect of EVs observed in AKI.
Zou et al. [28]	China	IRI (45 min on left kidney, remove right kidney)	SD rats	Male	C: 12 T: 12	UCMSC	211.4 ± 61.7^a^ (150–350)	Tail vein	After reperfusion	24 h	100 μg in 0.5 mL	MSC-EVs ameliorated renal ischaemic reperfusion injury by decreasing NK cells, and the spleen was not necessary in this process.
Zou et al. [29]	China	IRI (45 min on left kidney, remove right kidney)	Rats	Male	C: 18 T: 18	UCMSC	211.4 ± 61.7^a^ (150–350)	Tail vein	After reperfusion	24 h	100 μg in 1 mL	Human MSC-EVs protected against IRI-induced kidney injury through proangiogenesis effects in a HIF-1α-independent manner, and both the delivery of proangiogenesis-related VEGF and RNAs were involved in this process.
Zhang et al. [30]	China	IRI (45 min on left kidney, remove right kidney)	Rats	Male	C: 6 T: 6	WJMSC	30–500	Tail vein	After reperfusion	24 h	100 μg in 1 mL	MSC-EVs recovered AKI induced by IRI and helped balance oxidative stress/antioxidative responses to favourable levels by enhancing Nrf2/ARE activation.
Vinas et al. [31]	Canada	IRI (30 min, bilateral)	FVB mice	Male	C: 5 T: 7	UVEC	91 (40–100)^b^	Jugular vein	After reperfusion	24 h	20 μg	The delivery of UVEC exosomes reduced ischaemic kidney injury via the transfer of mir-486-5p targeting PTEN.
Shen et al. [32]	China	IRI (60 min, left kidney)	BALB/c mice	NR	C: 3 T: 3	BMSC	NR	Renal capsule	10 min after reperfusion	24 h	200 μg in 20 μL	CCR2 expressed on MSC-exo may play a key role in inflammation regulation and renal injury repair by acting as a decoy to suppress CCL2 activity.
Lin et al. [33]	Taiwan	IRI (bilateral)	SD rats	Male	C: 8 T: 8	ADMSC	NR	Intravenous	3 h after reperfusion	72 h	100 μg	Combined exosome-ADMSC therapy was superior to either one alone for protecting the kidney from acute IRI.
Gu et al. [34]	China	IRI (45 min on left kidney, remove right kidney)	SD rats	Male	C: 6 T: 6	WJMSC	NR	Tail vein	After reperfusion	24 h	100 μg in 1 mL	Single administration of WJMSC-EVs protected the kidney from IRI by inhibiting mitochondrial fission via mir-30.
de Almeida et al. [35]	Brazil	Cisplatin (15 mg/kg)	C57BL/6 mice	NR	C: 8 T: 8	Adult male mice inguinal adipose tissue	125	Intravenous	24 h after cisplatin administration	0, 24 h, 48 h, 72 h, 96 h	100 μg	MSCs regulated a particular miRNA subset of which mRNA targets were associated with the Wnt/TGF-β, fibrosis, and epithelial-mesenchymal transition signalling pathways. MSCs released MVs that transcriptionally reprogram injured cells, thereby modulating a specific miRNA-mRNA network.
Ju et al. [36]	China	IRI (60 min, left kidney)	SD rats	Male	C: 24 T: 24	UCMSC	142 (80–1000)^b^	Tail vein	After reperfusion	24 h, 48 h, 1 week, or 2 weeks	30 μg in 0.5 mL	MV-induced HGF synthesis in damaged tubular cells via RNA transfer facilitated cell dedifferentiation and growth, which are important regenerative mechanisms.
Burger et al. [37]	Canada	IRI (30 min, bilateral)	NOD-SCID mice	NR	C: 6 T: 7	UVEC	EV: 86 (40–100)^b^ MP: 223 (100–1000)^b^	Jugular vein	After reperfusion	24 h and 72 h	EVs: 15 μg UVECs: 10^6^ in 100 μL	UVEC-derived exosomes may mediate the protective response by inhibiting endothelial cell apoptosis.
Zou et al. [38]	China	IRI (60 min, left ischaemia, remove right kidney on day 12)	SD rats	Male	C: 18 T: 18	WJMSC	30–500	Tail vein	After reperfusion	24 h, 48 h, 2 weeks	100 μg in 1 mL	Single administration of MVs immediately after ischaemic AKI ameliorated renal injury in both the acute and chronic stages, and the anti-inflammatory property of MVs through the suppression of CX3CL1 may be a potential mechanism.
Zhang et al. [39]	China	IRI (60 min on left kidney, remove right kidney on day 12)	SD rats	Male	C: 6 T: 6	WJMSC	30–500	Tail vein	After reperfusion	24 h, 48 h, 2 weeks	100 μg in 1 mL	Single administration of WJMSC-MVs might protect the kidney by alleviating oxidative stress in the early stage of kidney IRI by suppressing NOX2 expression. Moreover, it reduced fibrosis and improved renal function.
Wang et al. [40]	China	IRI (45 min on left kidney, remove right kidney)	SD rats	Male	C: 6 T: 6	BMSC	30–60	Carotid artery	After reperfusion	48 h	100 μg	Rat BMSC-derived exosomes protected against IRI, with a decreased inflammatory response and apoptosis in rats.
Herrera Sanchez et al. [41]	Italy	Glycerol (8 mL/kg, 3 days)	SCID mice	NR	C: 18 T: 9	HLSC	174 ± 64	Tail vein	3 days after glycerol injection	Day 5 after glycerol administration	EVs produced by 3.5 × 10^5^ HLSCs	HLSCs increased recovery after AKI. EVs were the main component of HLSC-derived CM capable of promoting regeneration in experimental AKI.
Choi et al. [42]	Korea	IRI (30 min, bilateral)	FVB/N mice	Male	C: 5 T: 5	KMSC	NR	Tail vein	After reperfusion	0, 24 h, 72 h	2 × 10^7^ in 150 μL	KMSC-derived MPs may act as a source of proangiogenic signals and confer renoprotective effects in ischaemic kidneys.
Zhou et al. [43]	China	Cisplatin (6 mg/kg)	SD rats	Female	C: 6 T: 6	UCMSC	40–100	Renal capsule	24 h after cisplatin administration	1–5 days	200 μg	UCMSC-ex repaired cisplatin-induced AKI in rats and NRK-52E cell injury by ameliorating oxidative stress and cell apoptosis, promoting cell proliferation in vivo and in vitro.
Kilpinen et al. [44]	Finland	IRI (40 min, bilateral)	SD rats	Male	C: 8 T: 5	UCMSC	The smallest being around 20 nm and the largest > 500 nm	Left carotid artery	After reperfusion	0, 24 h, 48 h	NR	Inflammatory conditioning of MSCs influenced the protein content and functional properties of MVs, revealing the complexity of MSC paracrine regulation.
Cantaluppi et al. [45]	Italy	IRI (45 min on left kidney, remove right kidney)	Wistar rats	Male	C: 6 T: 6	EPCs were isolated from peripheral blood mononuclear cells	60–160	Tail vein	After reperfusion	48 h	30 μg	MVs derived from endothelial progenitor cells protected the kidney from ischaemic acute injury by delivering their RNA content, the miRNA cargo of which contributes to reprogramming hypoxic resident renal cells to a regenerative programme.
Bruno et al. [46]	Italy	Cisplatin (12 mg/kg)	SCID mice	Male	C: 8 T: 8	BMSC	135 (80–1000)^b^	Tail vein	8 h after cisplatin administration; 10, 14, and 18 after cisplatin	24 h	100 μg	MVs released from MSCs were found to exert a prosurvival effect on renal cells in vitro and in vivo, suggesting that MVs may contribute to renal protection conferred by MSCs by exerting a prosurvival effect on renal cells in vitro and in vivo, suggesting that MVs may contribute to renal protection.
Gatti et al. [47]	Italy	IRI (45 min on left kidney, remove right kidney)	SD rats	Male	C: 6 T: 6	BMSC	135 (80–1000)^b^	Intravenous	After reperfusion	48 h	30 μg	MVs released from MSCs protected from AKI induced by ischaemia reperfusion injury and from subsequent chronic renal damage.
Bruno et al. [48]	Italy	Glycerol (8 mL/kg, 3 days)	SCID mice	Male	C:6 T: 6	BMSC	180	Tail vein	3 days after glycerol injection	3 days, 5 days, 8 days, 15 days	15 μg	MVs derived from MSCs activated a proliferative programme in surviving tubular cells after injury via a horizontal transfer of mRNA.

*Abbreviations*: *ADMSC* adipose derived mesenchymal stromal cells, *AKI* acute kidney injury, *BMSC* bone marrow mesenchymal stromal cells, *C* control group, *CLP* caecal ligation and puncture, *CM* conditioned medium, *EPC* endothelial progenitor cell, *EVs* extracellular vesicles, *Gl-MSC* glomerular mesenchymal stromal cells, *HLSC* human liver stem cells, *IRI* ischaemia-reperfusion injury, *KMSC* kidney-derived mesenchymal stromal cells, *MP* microparticle, *MVs* microvesicles, *rIPC* remote ischaemic preconditioning, *NR* not reported, *SD* Sprague-Dawley, *T* treatment group, *UCMSC* umbilical cord mesenchymal stromal cells, *UVEC* umbilical vein endothelial cells, *WJMSC* Wharton’s jelly mesenchymal stromal cells

^a^Mean ± standard error

^b^Median (interquartile range)

### Quality assessment

All the included records were peer-reviewed publications, and all animals were allocated randomly to a treatment group and a control group; however, most studies did not report sample size calculation, blinded induction of the model, or blinded assessment of outcome. The details of the study quality assessment are shown in Additional file 1: Table S1.

### Primary outcome

**Fig. 2:**
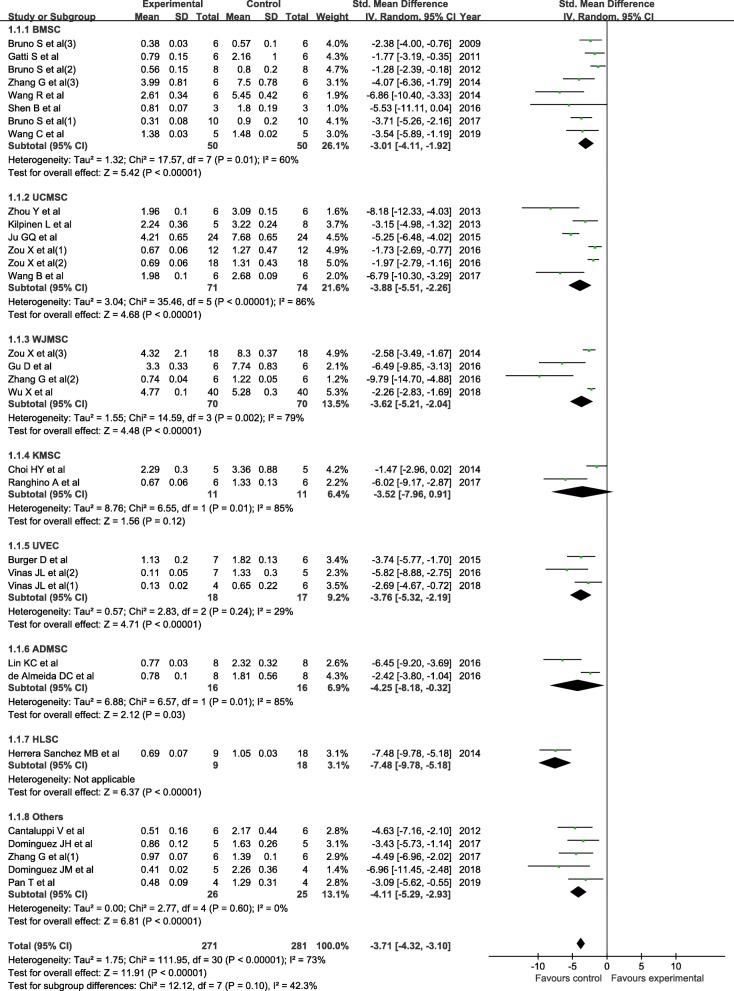
The forest plot shows the efficacy of EVs in reducing Scr levels in the AKI model. ADMSC, adipose-derived mesenchymal stromal cell; BMSC, bone marrow mesenchymal stromal cell; 95% CI, 95% confidence interval; EVs, extracellular vesicles; HLSC, human liver stem cell; IV, inverse variance; KMSC, kidney-derived mesenchymal stromal cell; Scr, serum creatinine; SD, standard deviation; UCMSC, umbilical cord mesenchymal stromal cell; UVEC, umbilical vein endothelial cell; WJMSC, Wharton’s jelly mesenchymal stromal cell

All studies reported the level of Scr. The pooled analysis showed that EVs can significantly reduce the Scr level when compared with the control (SMD = − 3.71; 95% CI = − 4.32, − 3.10; *P* < 0.01; *I*^2^ = 73%; Fig. 2). The subgroup analysis showed that all cell-derived exosomes are effective in reducing the Scr level (Fig. 2). The cumulative meta-analysis showed that the result did not change over time (Additional file 2: Figure S1). The sensitivity analysis showed that none of the single studies significantly influenced the result (Additional file 3: Figure S2). The multivariable meta-regression analysis showed that the delivery dose (*P* < 0.05) and cell origin of EVs (*P* < 0.05) were independent influential factors of SCr reduction.

### Secondary outcomes

**Fig. 3:**
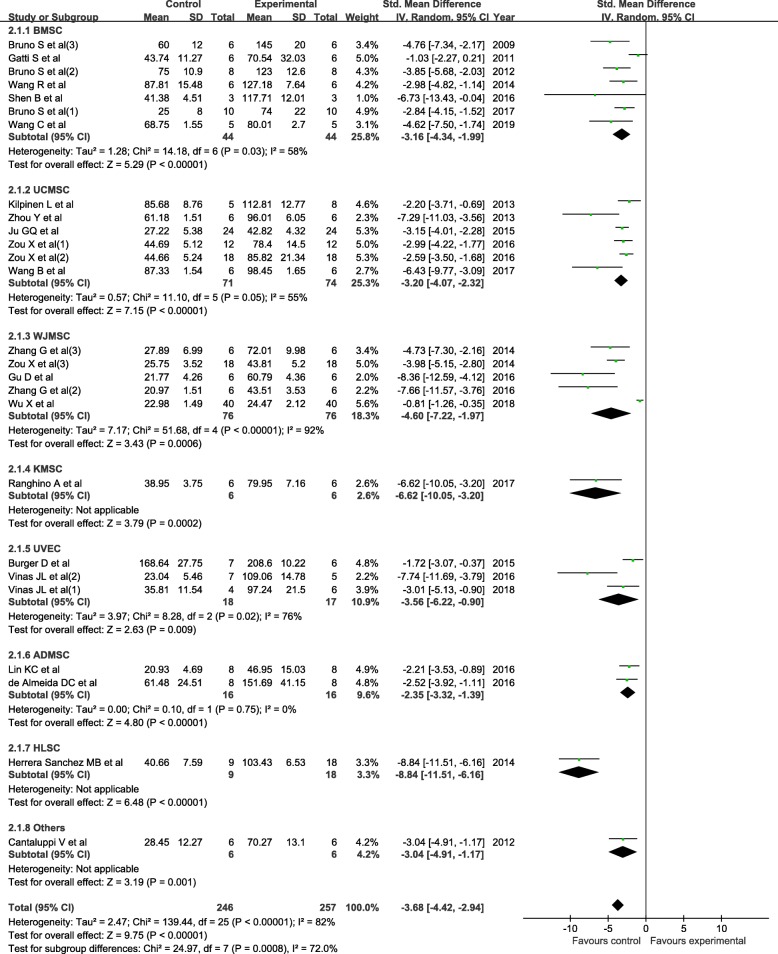
The forest plot shows the efficacy of EVs in reducing BUN levels in the AKI model. ADMSC, adipose-derived mesenchymal stromal cell; BMSC, bone marrow mesenchymal stromal cell; BUN, blood urea nitrogen; 95% CI, 95% confidence interval; EVs, extracellular vesicles; HLSC, human liver stem cell; IV, inverse variance; KMSC, kidney-derived mesenchymal stromal cell; SD, standard deviation; UCMSC, umbilical cord mesenchymal stromal cell; UVEC, umbilical vein endothelial cell; WJMSC, Wharton’s jelly mesenchymal stromal cell

The level of BUN was significantly decreased in the EV group (SMD = − 3.68; 95% CI = − 4.42, − 2.94; *P* < 0.01; *I*^2^ = 82%; Fig. 3). A subgroup analysis was performed according to the origin of the EVs, and the results indicated that all kinds of EVs included in this meta-analysis would reduce the level of BUN. The cumulative meta-analysis showed that the result did not change over time (Additional file 4: Figure S3). The sensitivity analysis showed that none of the single studies significantly influenced the result (Additional file 5: Figure S4). The meta-regression analysis showed that the cell origin of the EVs (*P* < 0.05) was an independent influential factor of BUN reduction.

Other secondary outcomes are summarised in Table 2. TUNEL assays were carried out in kidney tissue to detect apoptotic cells. Ten studies [20, 24, 30, 31, 34, 36, 38–40, 45] reported TUNEL results, and the pooled analysis showed that EVs can significantly reduce cell apoptosis. The tubular injury score was reported in six studies [21, 23, 30, 31, 33, 37], and the results showed that the injury score was lower in the EV group. In the EV group, the anti-inflammatory cytokine IL-10 [20, 24, 38] was significantly increased and the proinflammatory cytokine TNF-α [20, 35, 38] was significantly decreased (Table 2).

**Table 2:** Secondary outcomes

Outcomes	Number of studies	Std. mean difference (95%CI)	Test for effect (*P* value)	Heterogeneity, *I*^2^ (*P* value)
TUNEL	10 [20, 24, 30, 31, 34, 36, 38–40, 45]	− 6.25 (− 8.10, − 4.39)	< 0.01	87% (< 0.01)
Injury score	6 [21, 23, 30, 31, 33, 37]	− 3.90 (− 5.26, − 2.53)	< 0.01	54% (0.05)
IL-10	3 [20, 24, 38]	2.10 (1.18, 3.02)	< 0.01	68% (0.04)
TNF-α	3 [20, 35, 38]	− 2.65 (− 4.98, − 0.32)	0.03	95% (< 0.01)

*Abbreviations*: *IL* interleukin, *TNF* tumour necrosis factor

**Fig. 4:**
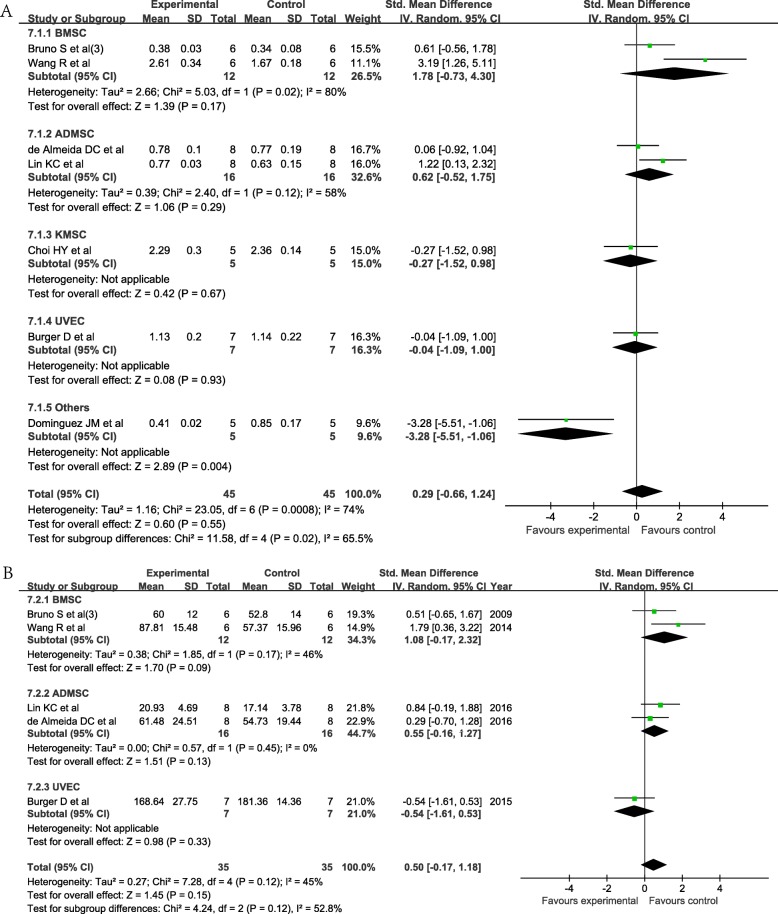
The forest plot compares the efficacy of cell-derived EVs with cells in the AKI model. **a** Forest plot of Scr. **b** Forest plot of BUN. ADMSC, adipose-derived mesenchymal stromal cell; BMSC, bone marrow mesenchymal stromal cell; BUN, blood urea nitrogen; 95% CI, 95% confidence interval; EVs, extracellular vesicles; IV, inverse variance; KMSC, kidney-derived mesenchymal stromal cell; Scr, serum creatinine; SD, standard deviation; UVEC, umbilical vein endothelial cell

Among the included studies, seven compared the efficacy of cell-derived EVs with cells in the AKI model. The results showed no significant difference in Scr (SMD = 0.29; 95% CI = − 0.66, 1.24; *P* = 0.55; *I*^2^ = 74%; Fig. 4a) or BUN (SMD = − 0.50; 95% CI = − 0.17, 1.18; *P* = 0.15; *I*^2^ = 45%; Fig. 4b) levels between the two groups. Meanwhile, no significant difference was found between stem cell-derived EVs and stem cells (Fig. 4).

### Publication bias

Significant publication bias was observed (*P* < 0.01; Additional file 6: Figure S5). We used the Trimfill method to recalculate the pooled risk estimation with the addition of missing studies (Additional file 7: Figure S6). However, the overall results were not significantly changed. Therefore, publication bias may have little effect on the meta-analysis outcomes (data not shown).

## Discussion

Our meta-analysis of 31 studies provided a comprehensive summary of the effect of EVs on the preclinical rodent AKI model. Pooled analyses confirmed that EV therapy could improve renal function and the inflammatory response status and reduce cell apoptosis in a preclinical rodent AKI model. The multivariable meta-regression analysis indicated that the delivery dose and cell origin of EVs were independent factors influencing the effect of EVs. Meanwhile, no significant difference was found between stem cell-derived EVs and stem cells. Therefore, the present meta-analysis provides important clues for human clinical trials on EVs.

A previous meta-analysis focused on this topic indicated that mesenchymal stromal cell-derived EVs produce a more marked therapeutic effect on recovery from renal failure than MSC-conditioned medium [49]. Our meta-analysis contained various types of cell-derived EVs and further evaluated the effect of EVs on cell apoptosis, the tubular injury score, and inflammatory cytokines, providing useful information for further clinical trials.

Many studies have shown that RNAs carried by EVs are the pivotal mechanism for their therapeutic function [11, 50], and the proteins contained in EVs are also related to many biological processes. EVs are membrane-bound vesicles released by all cell types, including stem/progenitor cells, which are important information carriers for regulating angiogenesis, extracellular matrix remodelling, gene expression, inflammation states, the cell cycle and proliferation, the phenotype of target cells, cell migration, and morphogenesis [51–54]. The surface molecules of EVs permit them to be targeted to recipient cells. Once attached to a target cell, EVs can induce signalling via a receptor-ligand interaction, be internalised by endocytosis and/or phagocytosis, or even fuse with the target cell’s membrane to deliver their content into its cytosol, thereby modifying the physiological state of the recipient cell [55, 56].

Compared with stem cells, stem cell-derived EVs have lower immunogenicity and may reduce some of the risks associated with cellular therapy, such as cytokine release syndrome [51]. In our meta-analysis, we demonstrated that stem cell-derived EVs were equally effective as stem cells when applied to treat AKI. In one study, MSC-derived EVs were superior to MSCs in reducing global renal damage levels in a rat model of donation after circulatory death (DCD) kidney [57]. Thus, EVs appear to be a promising approach for the repair of AKI.

The multivariable meta-regression analysis showed that the delivery dose and cell origin of EVs were independent factors influencing the efficacy of EVs. This suggests that we need to consider these factors when performing clinical trials. The properties and cargoes of EVs have been summarised in databases that are continuously updated, namely, Vesiclepedia, ExoCarta, and EVpedia [58]. Interestingly, the same cell may release EVs that differ in the content of their membrane lipid composition and in their intravesicular cargo [58, 59]. Therefore, further studies are urgently needed to explore the mechanism behind this phenomenon.

In our meta-analysis, various sizes of EVs were included. The large heterogeneity between EVs poses major obstacles to understanding the composition and functional properties of distinct secreted components [60]. One recent research reassessment of exosome composition established the differential distribution of protein, RNA, and DNA between small EVs and nonvesicular extracellular matter and demonstrated that small EVs are not vehicles of active DNA release [60]. It is important for further study to identify the key elements in AKI treatment.

One clinical trial tested the effects of MSC-derived EVs on the progression of chronic kidney disease (CKD) patients, and the results indicated that EVs can improve the estimated glomerular filtration rate (eGFR); decrease Scr, BUN, and TNF-α levels; and increase IL-10 levels [61]. However, significant translational challenges need to be addressed before the use of MSC-derived EVs for the clinical treatment of AKI. First, EV isolation and storage methods may potentially affect EV characteristics. It is challenging to ensure that recovered vesicles are truly from the extracellular space rather than from intracellular vesicles or artefactual particles released from cells broken during tissue harvest, processing (e.g. mechanical disruption), or storage (including freezing) [9]. Second, in most studies, the follow-up time ranged from 1 day to 2 weeks. Therefore, the long-term effects of EVs are a key issue that requires further exploration before their clinical application. Third, a development method that can be used to meet the large-scale clinical production requirement of a sufficient quantity of EVs is also a core problem [51]. Fourth, labelling EVs with lipophilic or surface-coating fluorophores may modify the physicochemical characteristics of EVs and alter the detection mode and/or uptake by target cells [9]; thus, the development of specific tracking tools is required to further detect EVs.

### Limitations

Several potential limitations to this meta-analysis should be considered. First, despite the fact that we performed subgroup and sensitivity analyses, the heterogeneity between studies cannot be remarkably reduced. This may weaken the stability of the results. Second, we included stem cell-derived EVs and other cell origin EVs, but we did not perform a direct comparison to identify the best option, which may have also increased the heterogeneity. Third, there was potential for the incomplete retrieval of identified research studies, which could have introduced publication bias. Finally, data extraction from graphics by using Engauge Digitizer software may have altered the original data, which would also affect the results.

## Conclusion

The present meta-analysis confirmed that EV therapy could improve renal function and the inflammatory response status and reduce cell apoptosis in a preclinical rodent AKI model. This provides important clues for human clinical trials on EVs.

## Supplementary information

**Additional file 1: Table S1.** Quality of eligible studies.

**Additional file 2: Figure S1.** Cumulative analysis of serum creatinine.

**Additional file 3: Figure S2.** Sensitivity analysis of serum creatinine.

**Additional file 4: Figure S3.** Cumulative analysis of blood urea nitrogen.

**Additional file 5: Figure S4.** Sensitivity analysis of blood urea nitrogen.

**Additional file 6: Figure S5.** Funnel plot of publication bias.

**Additional file 7: Figure S6.** Funnel plot of publication bias according to the Trimfill method.

## Data Availability

The authors confirm that all data underlying the findings are fully available without restriction. All relevant data are provided in the paper and its Additional files.

## Abbreviations

ADMSC: Adipose-derived mesenchymal stromal cell
AKI: Acute kidney injury
BMSC: Bone marrow mesenchymal stromal cell
BUN: Blood urea nitrogen
CM: Conditioned medium
EPC: Endothelial progenitor cell
EVs: Extracellular vesicles
HLSC: Human liver stem cell
IRI: Ischaemia-reperfusion injury
KMSC: Kidney-derived mesenchymal stromal cell
Scr: Serum creatinine
UCMSC: Umbilical cord mesenchymal stromal cell
UVEC: Umbilical vein endothelial cell
WJMSC: Wharton’s jelly mesenchymal stromal cell
