# Supplementary material for: Extracellular vesicles for acute kidney injury in preclinical rodent models: a meta-analysis
Source: Stem Cell Res Ther. 2020 Jan 3;11:11. doi: 10.1186/s13287-019-1530-4 (PMC6942291; doi:10.1186/s13287-019-1530-4)
Supplement: Supplementary file 1 — Additional file 1: Table S1. Quality of eligible studies. [file 13287_2019_1530_MOESM1_ESM.docx]

**Table S1** Quality of eligible studies.

| **Study** | **A** | **B** | **C** | **D** | **E** | **F** | **G** | **H** | **I** | **J** | **Total** |
| --- | --- | --- | --- | --- | --- | --- | --- | --- | --- | --- | --- |
| Wang C et al. 2019 [12] | **√** | **√** | **√** |  |  | **√** | **√** |  | **√** |  | 6 |
| Pan T et al. 2019 [13] | **√** | **√** | **√** |  |  | **√** | **√** |  | **√** | **√** | 7 |
| Wu X et al. 2018 [14] | **√** | **√** | **√** |  |  | **√** | **√** |  | **√** | **√** | 7 |
| Vinas JL et al. 2018 [15] | **√** | **√** | **√** |  |  | **√** | **√** |  | **√** |  | 6 |
| Dominguez JM et al. 2018 [16] | **√** | **√** | **√** |  |  | **√** | **√** |  | **√** |  | 6 |
| Zhang G et al. 2017 [17] | **√** | **√** | **√** |  |  | **√** | **√** |  | **√** | **√** | 7 |
| Wang B et al. 2017 [18] | **√** | **√** | **√** |  |  | **√** | **√** |  | **√** |  | 6 |
| Ranghino A et al. 2017 [19] | **√** | **√** | **√** |  |  | **√** | **√** |  | **√** |  | 6 |
| Dominguez JH et al. 2017 [20] | **√** | **√** | **√** |  |  | **√** | **√** |  | **√** | **√** | 7 |
| Bruno S et al. 2017 [21] | **√** | **√** | **√** |  |  | **√** | **√** |  | **√** |  | 6 |
| Zou X et al.2016 [22] | **√** | **√** | **√** |  |  | **√** | **√** |  | **√** |  | 6 |
| Zou X et al. 2016 [23] | **√** | **√** | **√** |  |  | **√** | **√** |  | **√** |  | 6 |
| Zhang G et al. 2016 [24] | **√** | **√** | **√** |  |  | **√** | **√** |  | **√** | **√** | 7 |
| Vinas JL et al. 2016 [25] | **√** | **√** | **√** |  |  | **√** | **√** |  | **√** |  | 6 |
| Shen B et al. 2016 [26] | **√** | **√** | **√** |  |  | **√** | **√** |  | **√** |  | 6 |
| Lin KC et al. 2016 [27] | **√** | **√** | **√** |  |  | **√** | **√** |  | **√** |  | 6 |
| Gu D et al. 2016 [28] | **√** | **√** | **√** |  |  | **√** | **√** |  | **√** | **√** | 7 |
| de Almeida DC et al. 2016 [29] | **√** | **√** | **√** |  |  | **√** | **√** |  | **√** | **√** | 7 |
| Ju GQ et al. 2015 [30] | **√** | **√** | **√** |  |  | **√** | **√** |  | **√** |  | 6 |
| Burger D et al. 2015 [31] | **√** | **√** | **√** |  |  | **√** | **√** |  | **√** |  | 6 |
| Zou X et al, 2014 [32] | **√** | **√** | **√** |  |  | **√** | **√** |  | **√** |  | 6 |
| Zhang G et al. 2014 [33] | **√** | **√** | **√** |  |  | **√** | **√** |  | **√** | **√** | 7 |
| Wang R et al. 2014 [34] | **√** | **√** | **√** |  |  | **√** | **√** |  | **√** |  | 6 |
| Herrera Sanchez MB et al. 2014 [35] | **√** | **√** | **√** |  |  | **√** | **√** |  | **√** |  | 6 |
| Choi HY et al. 2014 [36] | **√** | **√** | **√** |  |  | **√** | **√** |  | **√** |  | 7 |
| Zhou Y et al. 2013 [37] | **√** | **√** | **√** |  |  | **√** | **√** |  | **√** | **√** | 6 |
| Kilpinen L et al. 2013[38] | **√** | **√** | **√** |  |  | **√** | **√** |  | **√** |  | 6 |
| Cantaluppi V et al. 2012 [39] | **√** | **√** | **√** |  |  | **√** | **√** |  | **√** | **√** | 7 |
| Bruno S et al. 2012 [40] | **√** | **√** | **√** |  |  | **√** | **√** |  | **√** |  | 6 |
| Gatti S et al, 2011[41] | **√** | **√** | **√** |  |  | **√** | **√** |  | **√** |  | 6 |
| Bruno S et al, 2009[42] | **√** | **√** | **√** |  |  | **√** | **√** |  | **√** |  | 6 |

Abbreviation: A peer-reviewed journal; B temperature control; C animals were randomly allocated; D blind established model; E blinded outcome assessment; F use of anesthetic without significant intrinsic vascular protection activity; **G** , appropriate animal model (diabetic, advanced age or hypertensive); **H** calculation of sample size; **I** statement of compliance with animal welfare regulations; **J** statement of potential conflict of interests.
