# Supplementary figures and images for: Extracellular vesicles for acute kidney injury in preclinical rodent models: a meta-analysis
Source: Stem Cell Res Ther. 2020 Jan 3;11:11. doi: 10.1186/s13287-019-1530-4 (PMC6942291; doi:10.1186/s13287-019-1530-4)

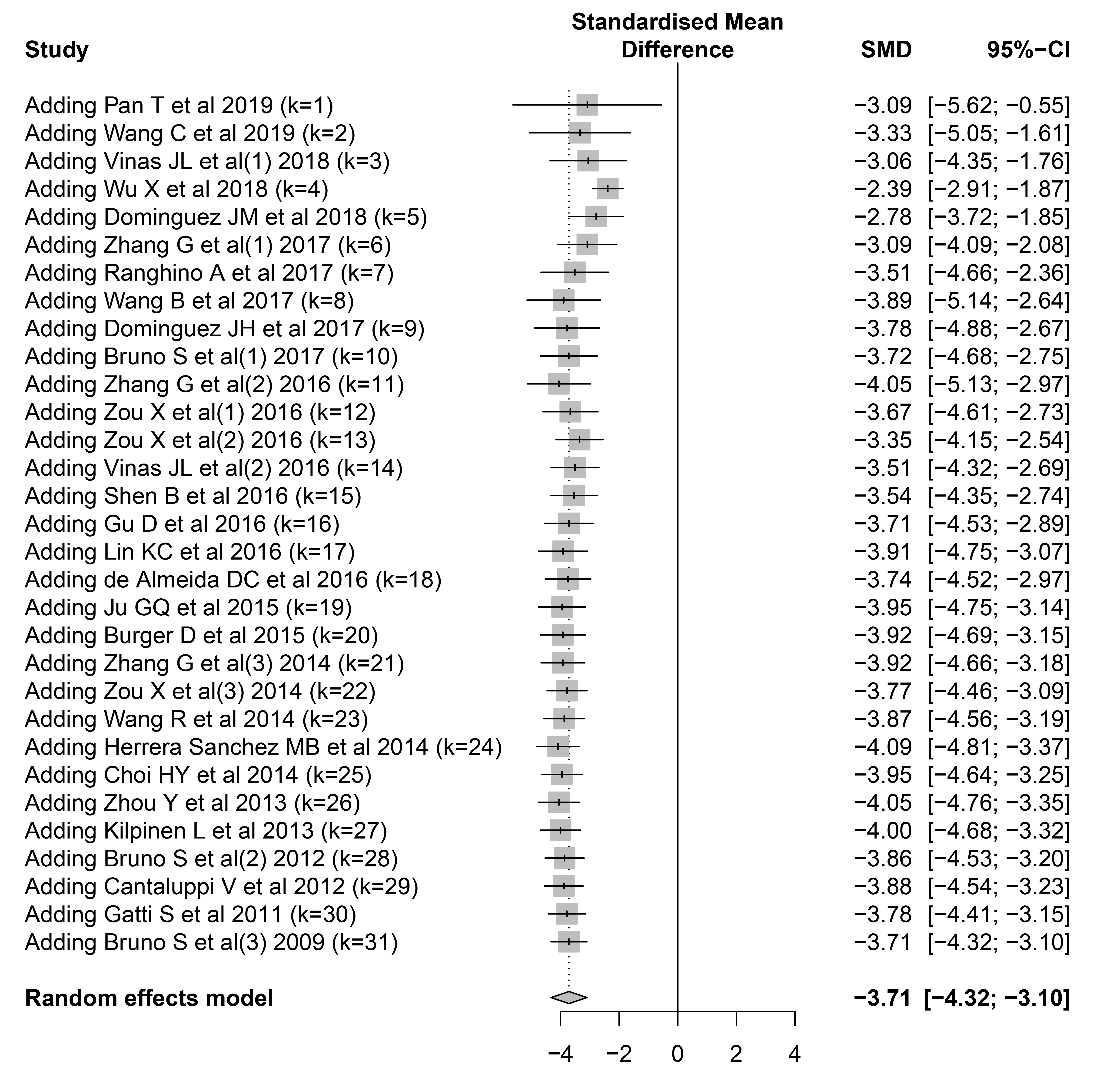

Supplement: Supplementary file 2 — Additional file 2: Figure S1. Cumulative analysis of serum creatinine. [file 13287_2019_1530_MOESM2_ESM.tif]

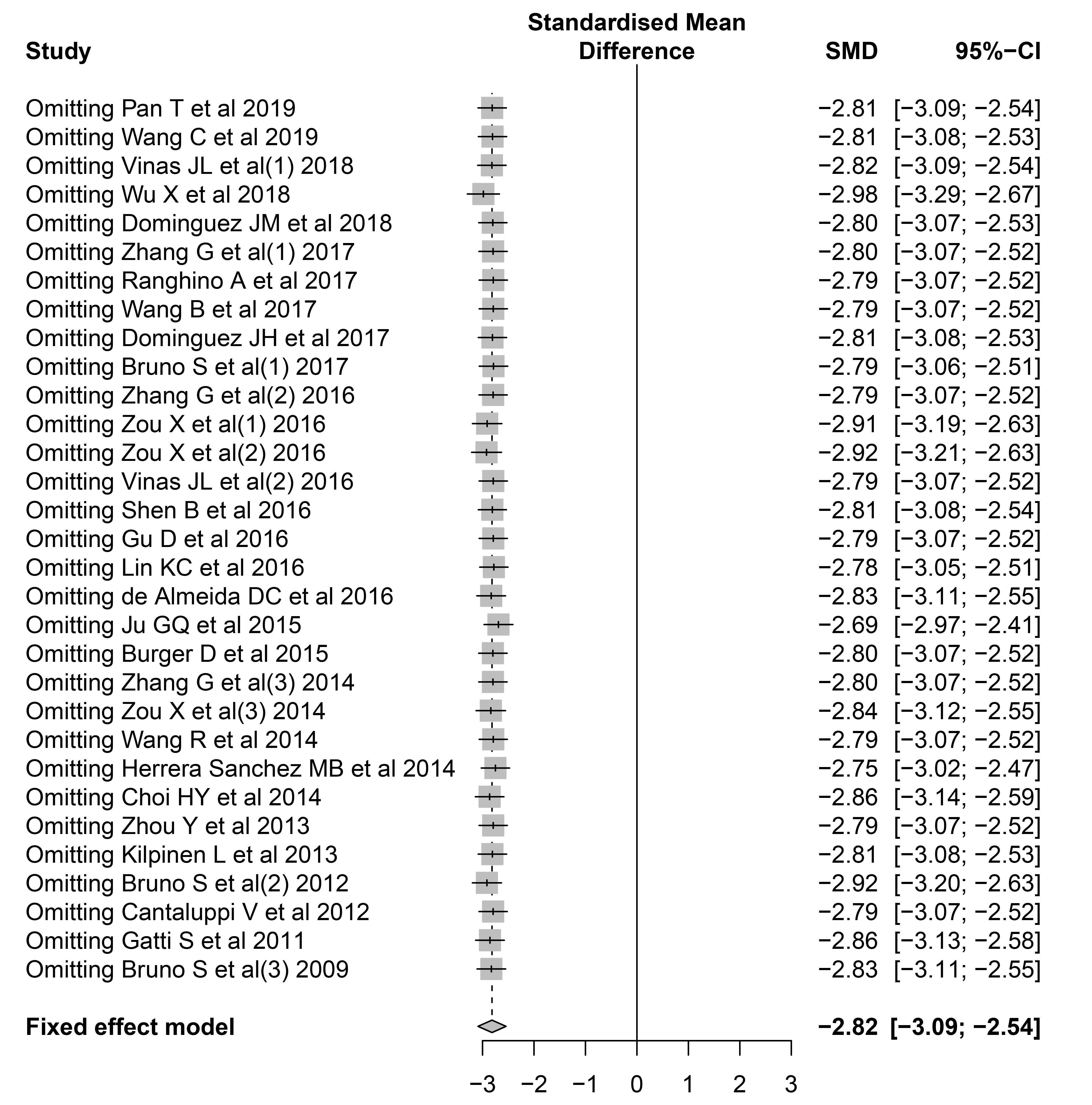

Supplement: Supplementary file 3 — Additional file 3: Figure S2. Sensitivity analysis of serum creatinine. [file 13287_2019_1530_MOESM3_ESM.tif]

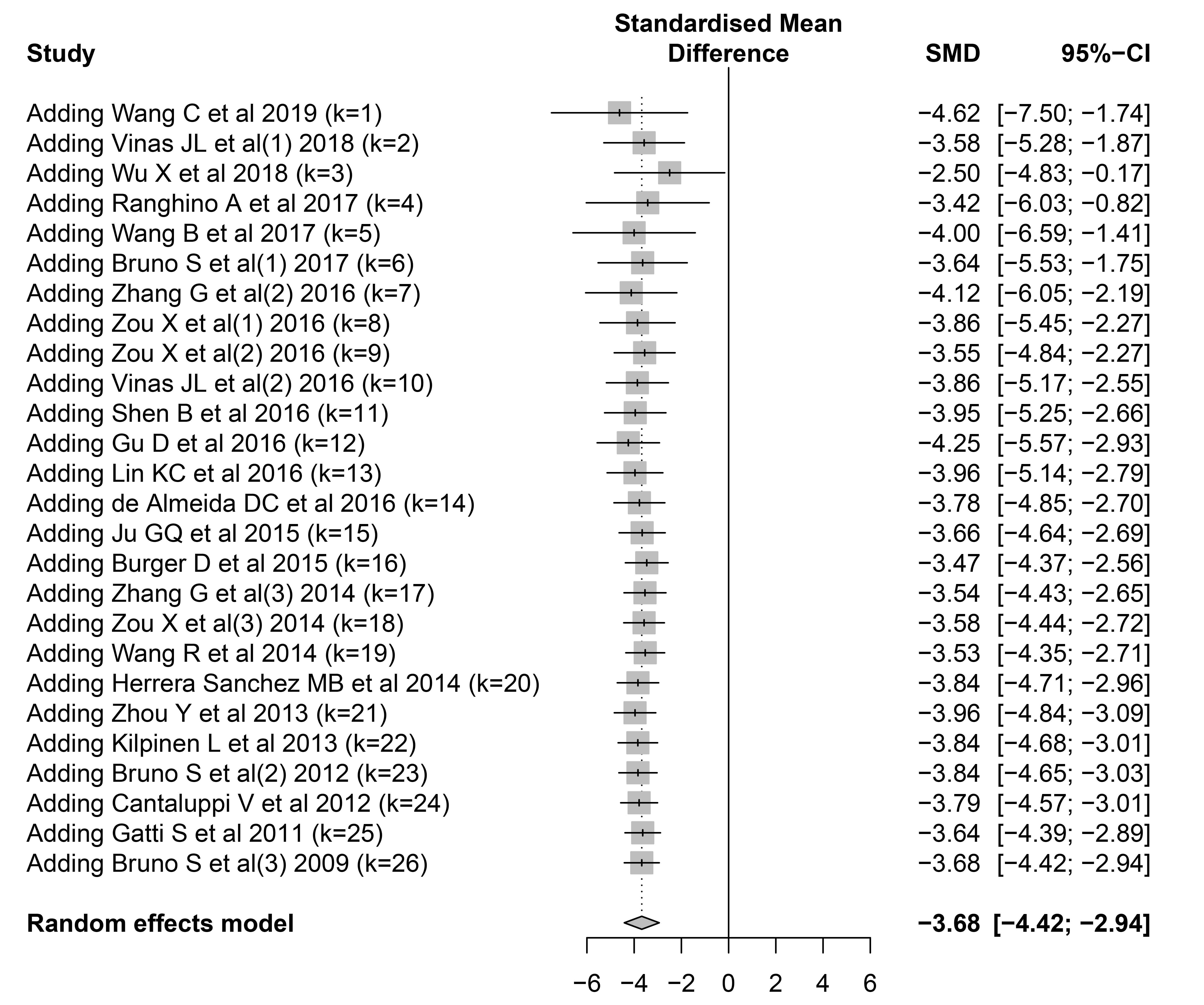

Supplement: Supplementary file 4 — Additional file 4: Figure S3. Cumulative analysis of blood urea nitrogen. [file 13287_2019_1530_MOESM4_ESM.tif]

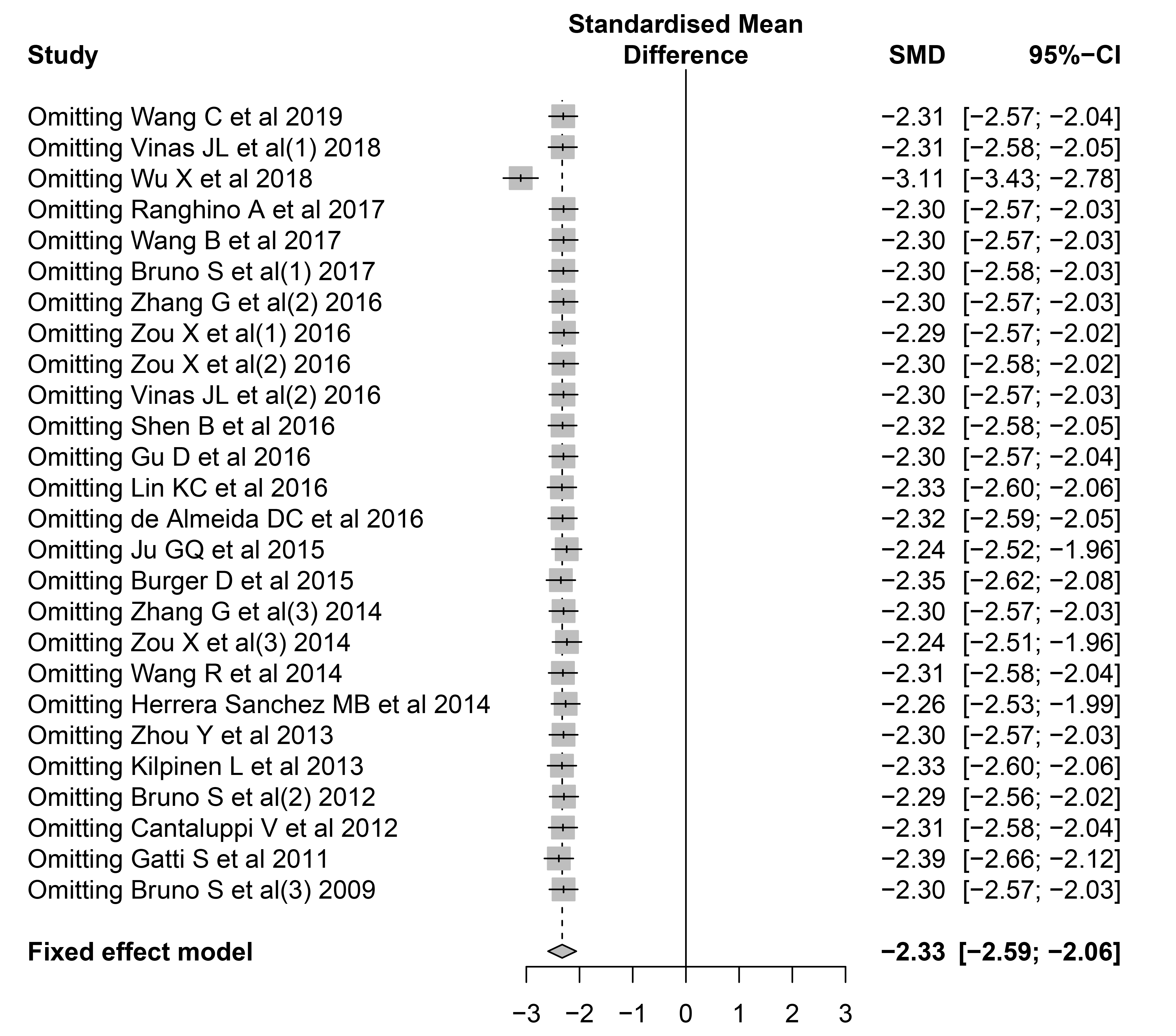

Supplement: Supplementary file 5 — Additional file 5: Figure S4. Sensitivity analysis of blood urea nitrogen. [file 13287_2019_1530_MOESM5_ESM.tif]

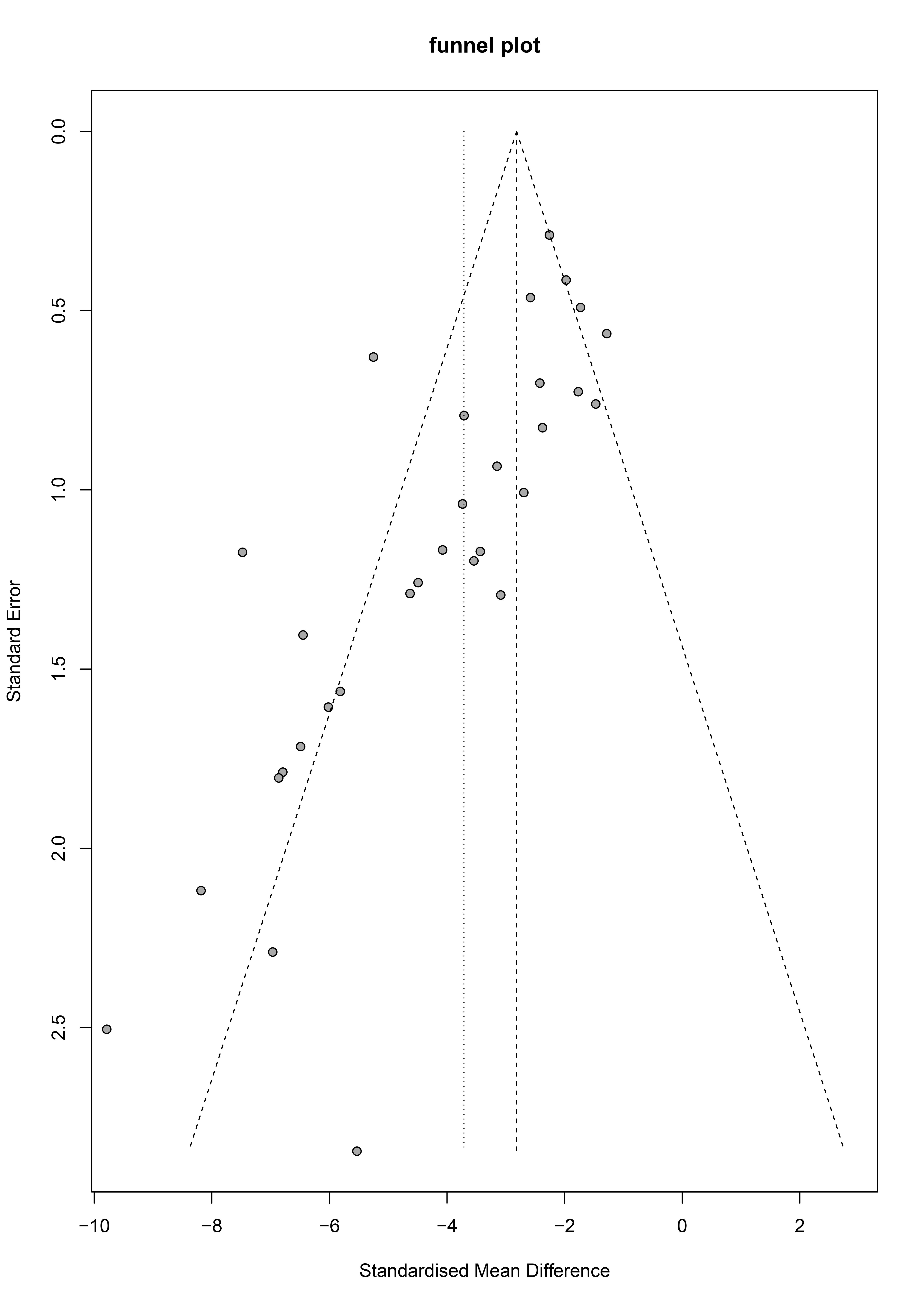

Supplement: Supplementary file 6 — Additional file 6: Figure S5. Funnel plot of publication bias. [file 13287_2019_1530_MOESM6_ESM.tif]

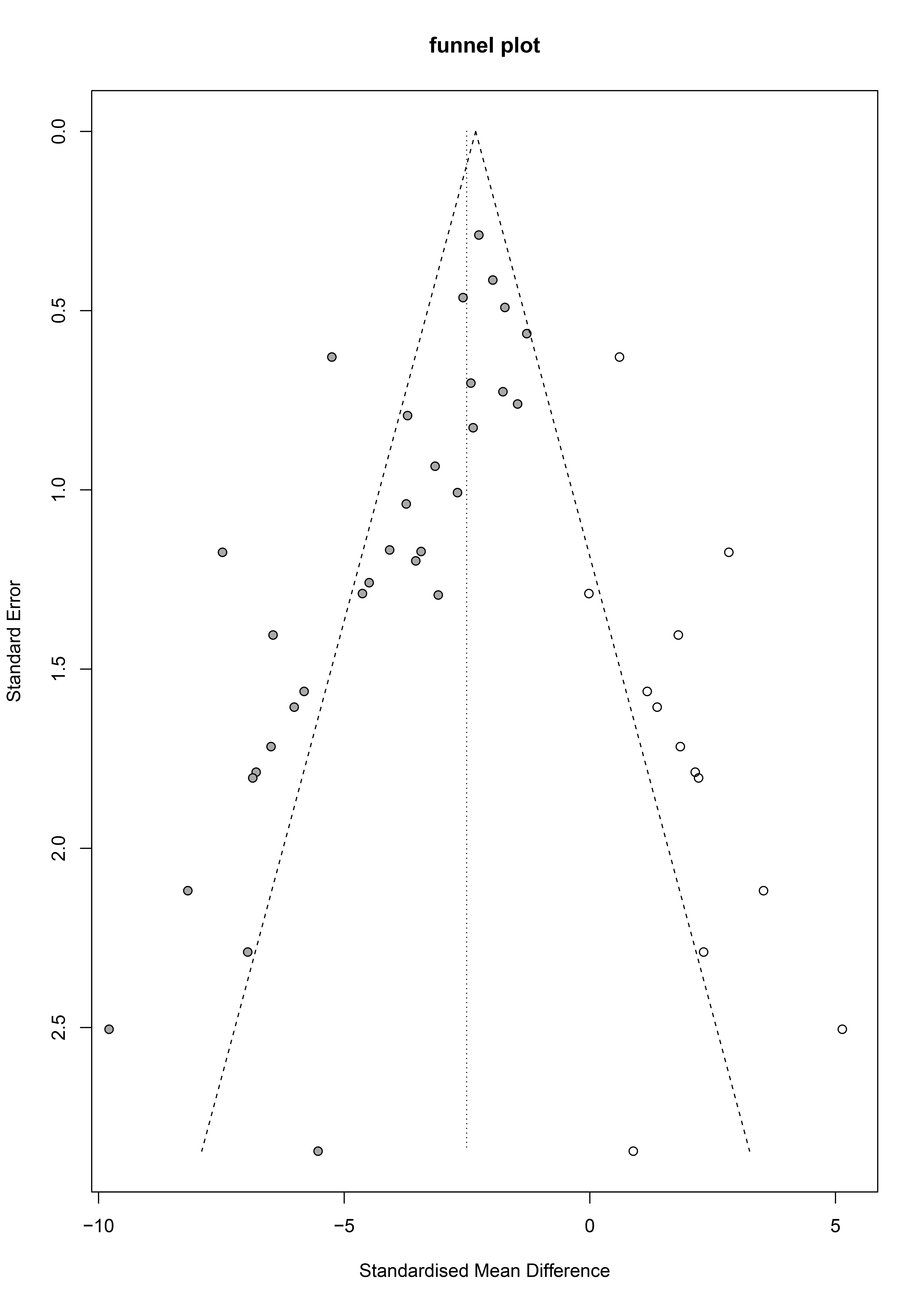

Supplement: Supplementary file 7 — Additional file 7: Figure S6. Funnel plot of publication bias according to the Trimfill method. [file 13287_2019_1530_MOESM7_ESM.tif]
